# Evolution of the folding landscape of effector caspases

**DOI:** 10.1016/j.jbc.2021.101249

**Published:** 2021-09-28

**Authors:** Suman Shrestha, A. Clay Clark

**Affiliations:** Department of Biology, University of Texas at Arlington, Arlington, Texas, USA

**Keywords:** caspase, protein folding, folding landscape, protein evolution, oligomerization, evolutionary biology, evolution, apoptosis, protease, ASA, accessible surface area, CA, common ancestor, PCP, procaspase, PDB, Protein Data Bank

## Abstract

Caspases are a family of cysteinyl proteases that control programmed cell death and maintain homeostasis in multicellular organisms. The caspase family is an excellent model to study protein evolution because all caspases are produced as zymogens (procaspases [PCPs]) that must be activated to gain full activity; the protein structures are conserved through hundreds of millions of years of evolution; and some allosteric features arose with the early ancestor, whereas others are more recent evolutionary events. The apoptotic caspases evolved from a common ancestor (CA) into two distinct subfamilies: monomers (initiator caspases) or dimers (effector caspases). Differences in activation mechanisms of the two subfamilies, and their oligomeric forms, play a central role in the regulation of apoptosis. Here, we examine changes in the folding landscape by characterizing human effector caspases and their CA. The results show that the effector caspases unfold by a minimum three-state equilibrium model at pH 7.5, where the native dimer is in equilibrium with a partially folded monomeric (PCP-7, CA) or dimeric (PCP-6) intermediate. In comparison, the unfolding pathway of PCP-3 contains both oligomeric forms of the intermediate. Overall, the data show that the folding landscape was first established with the CA and was retained for >650 million years. Partially folded monomeric or dimeric intermediates in the ancestral ensemble provide mechanisms for evolutionary changes that affect stability of extant caspases. The conserved folding landscape allows for the fine-tuning of enzyme stability in a species-dependent manner while retaining the overall caspase–hemoglobinase fold.

While folding landscapes of proteins have been studied for decades (1, 2, 3), many studies focused on small monomeric proteins or dimers with simple folding landscapes (4, 5). Studies of monomeric proteins have provided a wealth of information concerning the principles that govern intramolecular interactions during folding, but they do not consider intermolecular interactions provided by the interfaces of multimeric proteins (6). For some dimers, subunit interactions in the dimer interface lead to more complicated folding pathways when compared with simple two-state behavior involving only native dimer (N_2_) and unfolded monomers (U) (7, 8). Moreover, relatively little is known about the evolution of dimeric proteins compared with monomeric proteins (2, 9), although two-thirds of proteins form a multimeric assembly (10, 11). Thus, an understanding of the role of oligomerization in the folding landscape of a polypeptide sequence should include a consideration of the interface in assembly (12). In this regard, the caspase family of proteases is an attractive model system to study the evolution of protein folding landscapes. Caspases are a family of cysteinyl aspartate-specific proteases that initiate and execute programmed cell death and maintain cellular homeostasis in metazoans (13). There are two broad categories of caspases based on their function: inflammatory caspases (caspase-1, caspase-4, and caspase-5) and apoptotic caspases (Fig. 1*A*). The latter is further subdivided into two groups based on their entry into the apoptotic cascade. Initiator caspases (caspase-2, caspase-8, caspase-9, and caspase-10) act upstream and activate downstream effector procaspases (PCPs) (caspase-3, caspase-6, and caspase-7), which execute the cell death function (14). In addition, the caspase–hemoglobinase fold has been conserved for >650 million years (15), so the caspase family provides opportunities to examine the folding of monomers as well as changes to the folding landscape that resulted in oligomerization. As described by Aravind and Koonin (16), the term “caspase–hemoglobinase” refers to the conserved fold found in caspases, legumains (or hemoglobinases), paracaspases, metacaspases, and gingipains.

**Figure 1:**
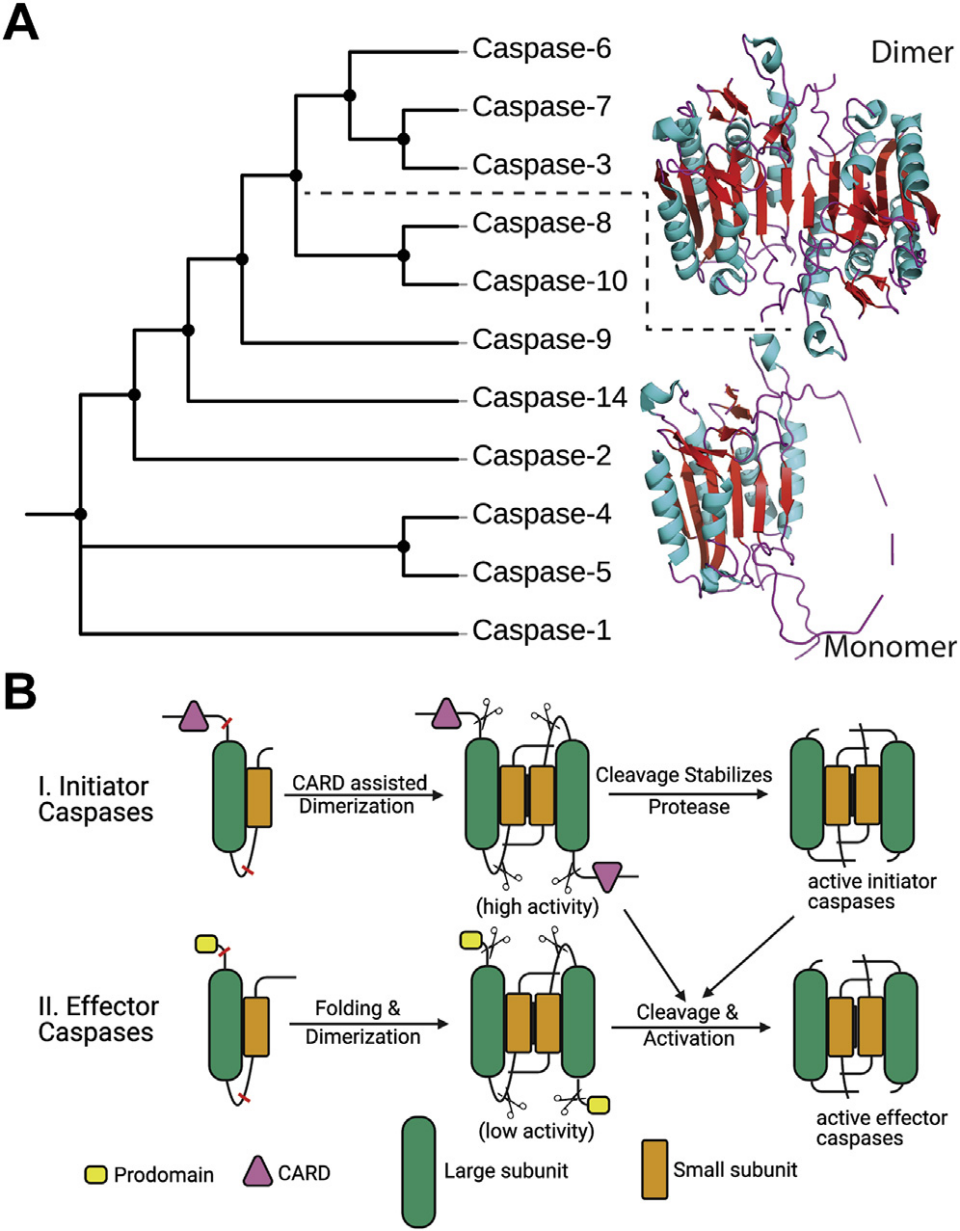
**Phylogenetic relationship and activation mechanism of apoptotic caspases.***A*, all caspases evolved from a common ancestor, then inflammatory (caspase-1, caspase-4, and caspase-5) and initiator caspases (caspase-8, caspase-9, caspase-10, caspase-14, and caspase-2) evolved as a monomer, whereas effector caspases (caspase-3, caspase-6, and caspase-7) evolved as dimers. *Solid lines* represent relationships to common ancestors and are not meant as evolutionary time lines. *B*, initiator caspase zymogens are stable monomers, and dimerization is sufficient for activation, whereas effector caspases are stable dimers that are activated by cleavage of an intersubunit linker. *Red lines* represent cleavage sites, and *scissors* represent cleavages.

More recently, evolutionary biochemists have used a new approach to study protein folding by examining the evolution of protein structure and function. Ancestral reconstruction methods enable one to use vertical comparisons (comparing ancestral to modern enzymes) as well as horizontal comparisons (comparing modern enzymes from multiple species) (17, 18, 19). The results of such studies potentially show the mechanisms by which changes in protein sequence have caused shifts in structure and function (20). The sequence determinants of protein structure and function and substitutions revealed by the evolutionary analysis in common evolutionary nodes can then be introduced singly or in combination into ancestral backgrounds. Ultimately, by examining the ancestral reconstructions and changes that occur throughout evolution of the protein, one can determine the effects of historical mutations on protein structure, function, and physical properties (19). We note that the data presented here describe the folding landscape of the effector caspases, and changes that occurred from the common ancestor (CA) to extant enzymes, rather than the phylogenetic relationship. In contrast, the phylogenetic tree in Figure 1*A* shows the evolutionary events leading to each caspase, and the lines in the figure are meant only to illustrate the caspase subfamilies. Accordingly, the line lengths in Figure 1*A* do not represent evolutionary time lines.

The effector and initiator caspases evolved by gene duplication and divergence from a CA more than 650 million years ago (Fig. 1*A*) (15). The CA provided a scaffold for the modern caspases to acquire distinct properties, such as formation of oligomers, changes in stability, enzyme specificity, and allosteric regulation (15). All caspases are produced in the cell as inactive zymogens that must be activated during the apoptotic cascade. In general, initiator PCPs are stable monomers, and dimerization is sufficient for activation, whereas effector PCPs are stable, yet inactive dimers, and are activated *via* cleavage by initiator caspases (Fig. 1*B*). The oligomeric form of the zymogen and its activation mechanism is key to regulating apoptosis (13, 15). While the amino acid sequence identity is low between caspase subfamilies (∼40%) (15), the caspase–hemoglobinase fold is well conserved (21) even from distantly related species of vertebrates and invertebrates, such as human, *Danio rerio*, *Caenorhabditis elegans*, *Drosophila melanogaster*, and *Porites astreoides* (22, 23, 24, 25, 26). The structure of the PCP monomer is characterized by a six-stranded β-sheet core with several α-helices on the surface (6). Each monomer of the PCP homodimer consists of approximately 300 amino acids organized into an N-terminal prodomain followed by a protease domain. The protease domain is further divided into large and small subunits that are connected by a short intersubunit linker (Fig. 1*B*).

Previously, we showed that the human PCP-3 dimer assembles by a four-state equilibrium mechanism in which the unfolded protein partially folds to a monomeric intermediate. Following dimerization of the intermediate, the protein undergoes a conformational change to form the native dimer (2U ⇄ 2I ⇄ I_2_ ⇄ N_2_) (27). Dimerization results in a substantial increase in conformational free energy, Δ*G*°_conf_, for the dimer (∼25 kcal mol^−1^) *versus* the monomer (∼7 kcal mol^−1^) at 25 °C (27). Furthermore, by examining the changes in Δ*G*°_conf_ *versus* pH, we showed that the PCP-3 dimer dissociates at lower pH because of a pair of histidine residues that contribute to salt bridges across the dimer interface (28). Thus, our previous data showed that the per-residue contribution to the total conformational free energy of the dimer (Δ*G*°_conf_) increases from 0.025 kcal/mol/amino acid to 0.044 kcal/mol/amino acid (27, 28). However, because human PCP-3 is the only caspase in which the folding properties have been examined, the evolutionary trajectories that resulted in dimer formation remain unknown.

We recently used ancestral protein reconstruction techniques to resurrect the CA of the caspase-3/6/7 subfamily (29). In those studies, we examined the robustness of reconstruction methods by resurrecting two sequences (called AncCP-Ef1 and AncCP-Ef2) from the pool of possible sequences of CA, and we characterized the proteins biochemically and structurally (15). Here, we examine the evolution of the caspase folding landscape using the ancestral reconstruction AncCP-Ef2 (referred to here as PCP-CA). The results are compared with those for extant human PCP-6 and PCP-7 as well as our previous studies for PCP-3 (27, 28). We examined urea-induced equilibrium unfolding over a broad pH range to compare the folding pathways of all three effector caspase subfamilies. The data show that the caspase folding landscape was first established and then retained for >650 million years. The CA PCP-CA forms a weak dimer, and the dimer was stabilized early in the evolution of the subfamily. Of the three extant human effector caspases, caspase-6 is the most stable, whereas caspase-7 is the least stable. The folding landscape of PCP-7 is more similar to that of the CA, which is consistent with previous phylogenetic data that show caspase-7 is closest to the CA (15). In PCP-3 and PCP-6, folding intermediates were stabilized later in evolution.

## Results

A protomer of PCP-6, PCP-7, and PCP-CA consists of 293, 303, and 275 amino acids, respectively (Table S1). Under some conditions, such as the high protein concentrations found in heterologous expression systems, effector caspase activation is autocatalytic. In order to prevent autoproteolysis during expression in *Escherichia coli*, we substituted the active site cysteine with a serine residue for our equilibrium unfolding studies, as described previously for PCP-3 (27).

**Figure 2:**
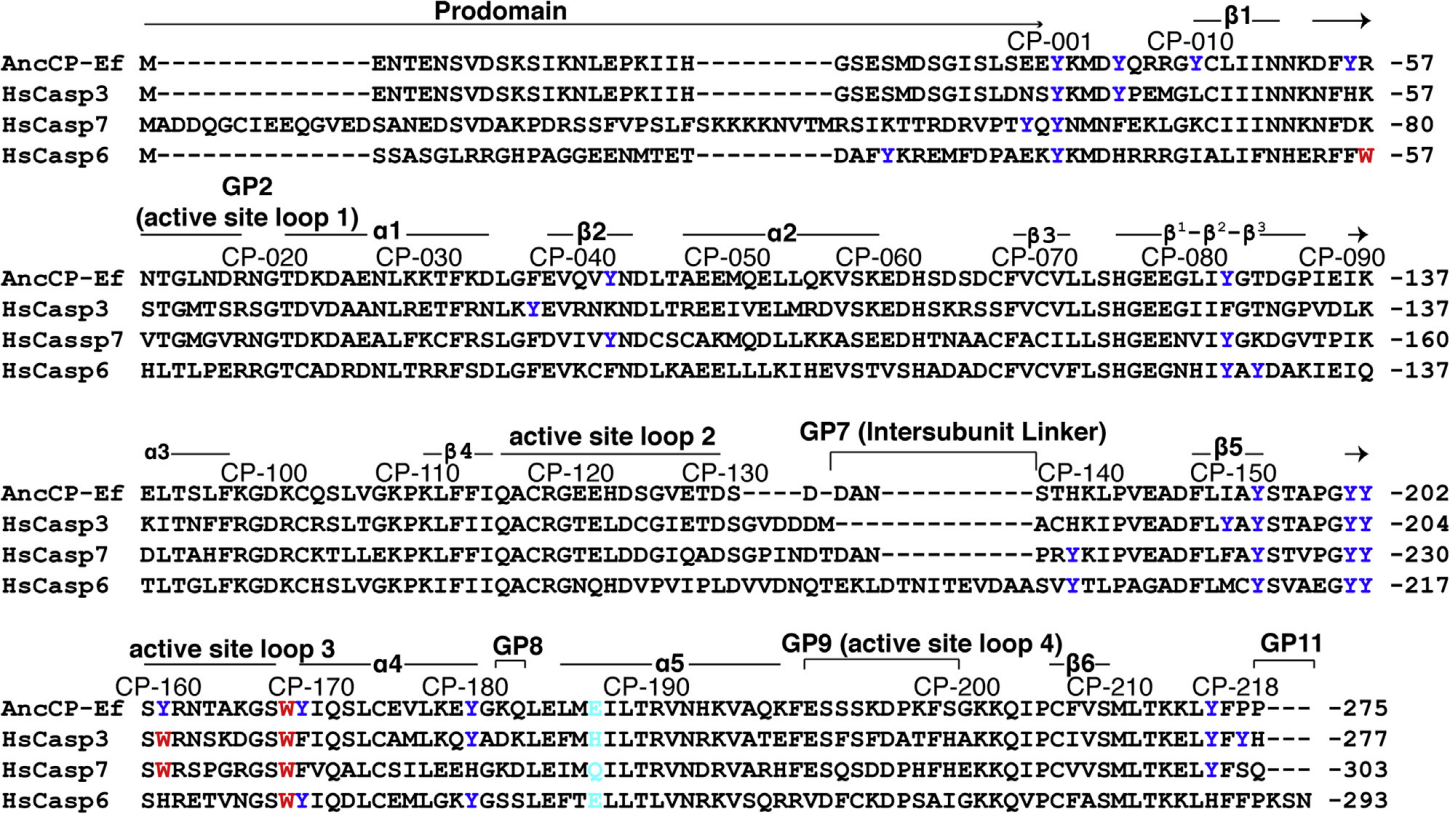
**Multiple sequence alignment of human effector caspases with a common ancestor.** CP refers to common position number of caspases (54), and individual sequence number is indicated at the right side of sequences. Secondary structural elements are indicated, and tyrosine residues (*blue*), tryptophan residues (*red*), and CP-186 (*cyan*), unique amino acid among effector caspases in dimeric interface are highlighted.

**Figure 3:**
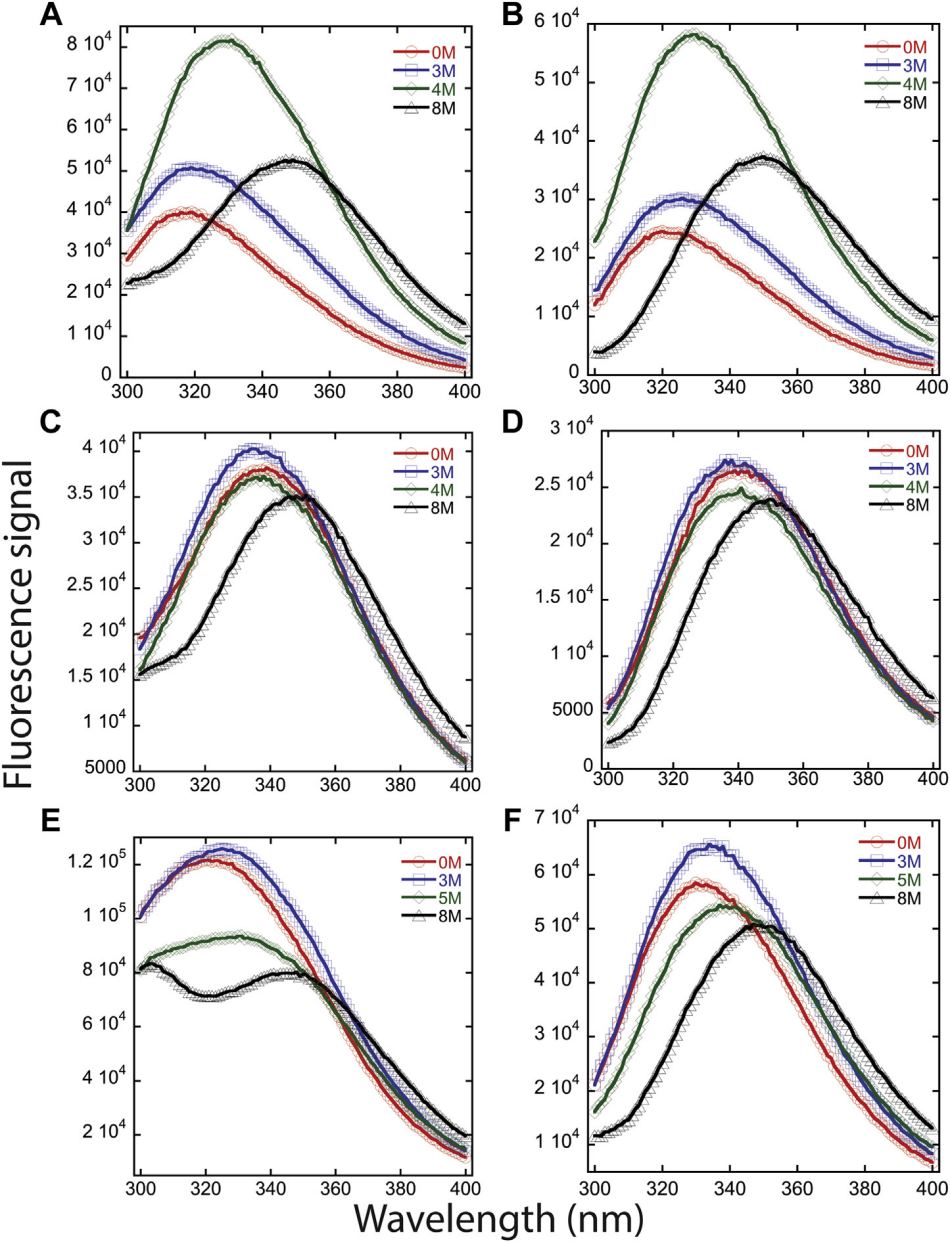
**Fluorescence emission spectra of effector caspases following excitation.***Left*, excitation at 280 nm or right; *right*, 295 nm. Emission spectra of PCP-6 (*A* and *B*), PCP-7 (*C* and *D*), and PCP-CA (*E* and *F*) at 2 μM protein concentration in a buffer of 20 mM phosphate, pH 7.5, containing urea (displayed in inset of each graph). PCP, procaspase.

PCP-6 and PCP-7 have two tryptophan residues, whereas PCP-CA has only one tryptophan (Fig. 2 and Table S1). In PCP-6, one tryptophan resides in active site loop 1 (Fig. S1), whereas the second tryptophan resides in active site loop 3. In PCP-7 and PCP-CA, the tryptophans are found only in active site loop 3 (Figs. 2 and S1). PCP-6, PCP-7, and PCP-CA have 10, 9, and 13 tyrosine residues, respectively, and they are well distributed in the primary sequence (Figs. 2 and S1). Native PCP-6 has a fluorescence emission maximum at 319 nm (Fig. 3, *A* and *B*), whereas that of native PCP-7 is at 338 nm, when excited either at 280 nm or 295 nm (Fig. 3, *C* and *D*). In the case of a PCP-CA, the fluorescence emission maximum is 321 nm when excited at 280 nm and 330 nm when excited at 295 nm (Fig. 3, *E* and *F*). Overall, the data show that the tryptophan residues in PCP-7 are more solvent exposed than those of PCP-6 or of PCP-CA. In phosphate buffer containing 8 M urea, the fluorescence emission maximum is red shifted to ∼350 nm following excitation at 280 nm or 295 nm in all three proteins, indicating that the proteins were largely unfolded under these solution conditions (Fig. 3). At intermediate concentrations of urea (3–5 M), the emission maxima were red shifted in the case of PCP-6 and PCP-CA but were largely unaffected in the case of PCP-7. We note that, in the unfolded state, PCP-CA exhibits two peaks when excited at 280 nm and a single peak when excited at 295 nm. When excited at 280 nm, but not at 295 nm, the two peaks begin to separate when PCP-CA is incubated in 4.5 M urea and are fully separated by 6 M urea (data not shown). As described previously by Lakowicz (30), the two peaks likely represent ionized and nonionized tyrosinyl residues, whereas the tryptophanyl residues contribute to the fluorescence emission at ∼350 nm in the unfolded protein. The changes in emission maxima are described more fully later.

### Equilibrium unfolding of extant and ancestor caspases

**Figure 4:**
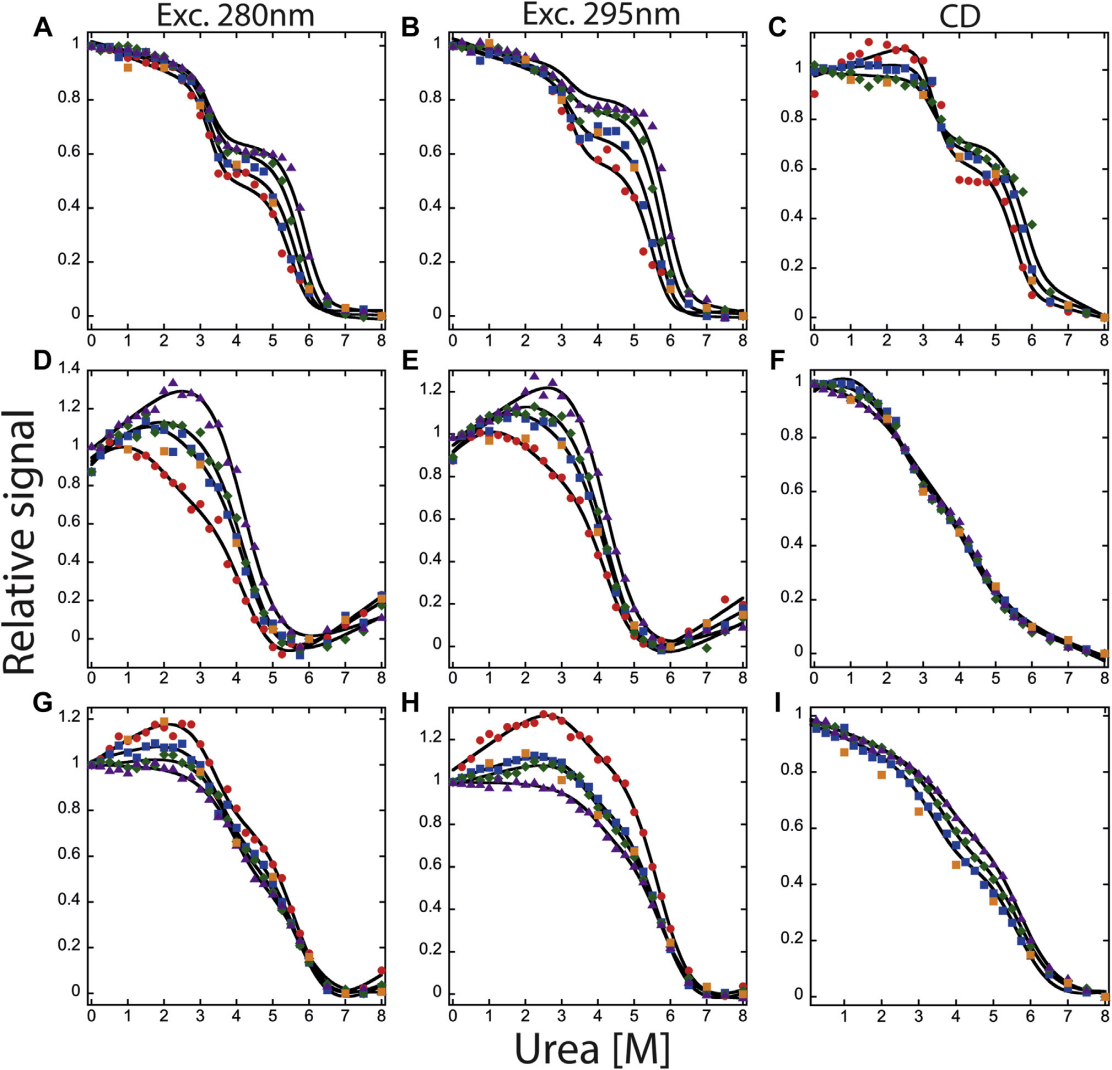
**Equilibrium unfolding of effector caspases at pH 7.5.** Equilibrium unfolding of PCP-6 (*A*, *B*, and *C*), PCP-7 (*D*, *E*, and *F*), and PCP-CA (*G*, *H*, and *I*) monitored by fluorescence emission with excitation at 280 nm (*left column*), 295 nm (*middle column*), and CD (*right column*). Four different protein concentrations were used to measure unfolding monitored by fluorescence emission, and three different protein concentrations were used in to monitor unfolding by CD. *Colored solid symbols* represent raw data, and corresponding *solid lines* represent the global fits of the data to an appropriate model as described in the text. The following protein concentrations were used: 0.5 μM (), 1 μM (), 2 μM (), and 4 μM (). *Orange squares* () represent refolding data of 1 μM protein to show reversibility. PCP, procaspase.

Changes in the fluorescence emission and CD properties of PCP-3 as a function of urea concentration have been described previously (27, 28). In this study, we examined the equilibrium unfolding of PCP-6, PCP-7, and PCP-CA at pH 7.5 as a function of urea concentration (0–8 M), and the results are shown in Figure 4. Renaturation experiments of all three proteins demonstrated that the folding transitions are reversible.

For PCP-6 at pH 7.5, both the fluorescence emission data (Fig. 4, *A* and *B*) as well as the CD data (Fig. 4*C*) show little to no change in signal between 0 and ∼2.5 M urea. One then observes a cooperative decrease in the signal between ∼3.5 and 5 M urea, demonstrating a plateau between the native and unfolded signals. A second cooperative transition occurs between ∼5 and 6.5 M urea. For PCP-6, the relative signal of the plateau as well as the second cooperative transition is dependent on the protein concentration.

In contrast to PCP-6, both PCP-7 (Fig. 4, *D*–*F*) and PCP-CA (Fig. 4, *G*–*I*) show a protein concentration–dependent increase in the relative signal between 0 and ∼3 M urea, followed by a cooperative decrease in the signal between ∼3 and 6 M urea to form the unfolded state. Thus, the unfolding data are similar for PCP-7 and PCP-CA, where the protein concentration dependence is observed in the first unfolding transition rather than the second transition, as in the case of PCP-6.

Altogether, the data in Figure 3, Figure 4 show that the three proteins fold through a stable intermediate, which can be characterized by a red shift in fluorescence emission and a concomitant loss of secondary structure compared with the native dimer. In the case of PCP-6, however, the protein concentration dependence is observed in the second transition, whereas both PCP-7 and PCP-CA show a protein concentration dependence in the first transition. Overall, the data suggest that while the partially folded intermediates that form during urea-induced unfolding have more solvent-exposed tryptophans and less secondary structure, compared with the native dimer, the intermediate remains dimeric in the case of PCP-6, whereas the intermediate is monomeric in the cases of PCP-7 and PCP-CA.

### Global fitting of equilibrium unfolding data

The experimental design described previously at pH 7.5 for monitoring fluorescence emission (three to four protein concentrations, each with two excitation wavelengths) and CD (three protein concentrations) provides 11 datasets for each protein are fit globally to determine the free energy and the cooperativity index (*m* value) for each unfolding transition. In the case of PCP-6, the data were best fit to the three-state equilibrium model described in Equation 1. In this model, the dimeric native conformation, N_2_, isomerizes to a dimeric intermediate, I_2_, and the dimeric intermediate dissociates and unfolds to monomers. The dissociation of I_2_ to 2U leads to a protein concentration–dependent change in the midpoint of the second transition, as shown in Figure 4, *A*–*C*. Based on this model, we have determined the conformational free energy, Δ*G*°_conf_, and the *m* values for each step of unfolding. The solid lines in Figure 4, *A*–*C* are the results of global fits of the model to the data. The free energy change, $\Delta G_{1}^{{\text{H}}_{2}\text{O}}\;$and the cooperativity index, m_1_, for the first step of unfolding, the isomerization of N_2_ to I_2_, are 8.4 ± 0.8 kcal/mol and 2.6 ± 0.3 kcal mol^−1^ M^−1^, respectively (Table 1). The free energy change, $\Delta G_{2}^{{\text{H}}_{2}\text{O}}$, and cooperativity index, m_2_, for the dissociation and complete unfolding of the dimeric intermediate to two unfolded monomers (I_2_ ⇄ 2U) are 24.4 ± 0.9 kcal/mol and 2.9 ± 0.2 kcal mol^−1^ M^−1^, respectively. Overall, the data demonstrate that PCP-6 is very stable, with the total conformational free energy of 32.8 kcal/mol at pH 7.5 and 25 °C.

**Table 1:** Thermodynamic parameters of each step of folding/unfolding of extant and ancestral effector caspases at pH 7.5

Proteins	Equilibrium mechanism	Free energy changes (Δ*G*°_conf_) (kcal mol^−1^)	Cooperativity index (*m* value) (kcal mol^−1^ M^−1^)	Total Δ*G*°_conf_ (kcal mol^−1^)	*m*_total_ (kcal mol^−1^ M^−1^)
PCP-3^a^	N_2_ ⇌ I_2_	7.9 ± 1.3	2.8 ± 0.5	24.8 ± 0.9	4.5 ± 0.7
	I_2_ ⇌ 2I	9.7 ± 1.0	0.5 ± 0.1		
	2I ⇌ 2U	7.2 ± 0.5	1.2 ± 0.1		
PCP-6	N_2_ ⇌ I_2_	8.4 ± 0.8	2.6 ± 0.3	32.8 ± 1.5	5.5 ± 0.5
	I_2_ ⇌ 2U	24.4 ± 0.9	2.9 ± 0.2		
PCP-7	N_2_ ⇌ 2I	10.2 ± 0.2	1.3 ± 0.1	15.4 ± 0.3	2.5 ± 0.2
	2I ⇌ 2U	5.2 ± 0.1	1.2 ± 0.1		
PCP-CA	N_2_ ⇌ 2I	14.9 ± 0.1	2 ± 0.1	21.8 ± 1.3	3.2 ± 0.2
	2I ⇌ 2U	6.9 ± 0.3	1.2 ± 0.1		

a Data from Bose and Clark (27).

For PCP-7 and PCP-CA, the global fits demonstrate that the data are well described by a three-state equilibrium model. In contrast to PCP-6, described previously, the partially folded intermediate is monomeric for PCP-7 and PCP-CA (Equation 2). The solid lines in Figures 4, *D*–*F* (PCP-7) and 4*G*–*I* (PCP-CA) are the results of fits of the data to the model. In the case of PCP-7, the free energy change, $\Delta G_{1}^{{\text{H}}_{2}\text{O}}$, and the cooperativity index, m_1_, for the first step of unfolding, the dissociation of N_2_ to 2I, are 10.2 ± 0.2 kcal/mol and 1.3 ± 0.1 kcal mol^−1^ M^−1^, respectively. The free energy change, $\Delta G_{2}^{{\text{H}}_{2}\text{O}}$, and cooperativity index, m_2_, for the complete unfolding of the monomeric intermediate to unfolded monomeric proteins (I ⇄ U) are 5.2 ± 0.1 kcal/mol and 1.2 ± 0.1 kcal mol^−1^ M^−1^, respectively (Table 1). Similarly, for PCP-CA, $\Delta G_{1}^{{\text{H}}_{2}\text{O}}$ and m_1_ are 14.9 ± 0.1 kcal/mol and 2.0 ± 0.1 kcal mol^−1^ M^−1^, respectively. For unfolding of the monomeric intermediate of PCP-CA (I ⇄ U), $\Delta G_{2}^{{\text{H}}_{2}\text{O}}$ and m_2_ are 6.9 ± 0.3 kcal/mol and 1.2 ± 0.1 kcal mol^−1^ M^−1^, respectively (Table 1).

**Figure 5:**
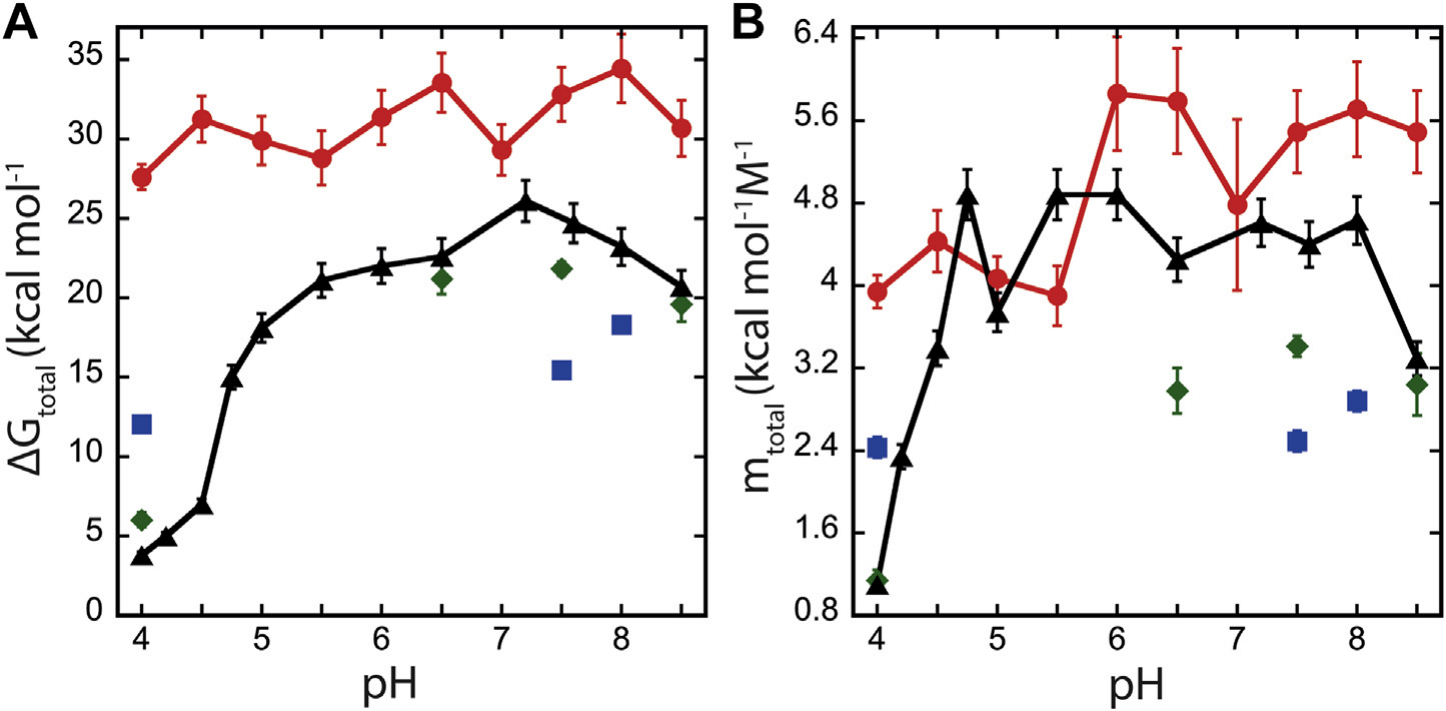
**Conformational free energy as a function of pH.** Comparison of total conformational free energy (Δ*G*_total_) (*A*) and total *m* value (*m*_total_) (*B*) changes as a function of pH between PCP-6 (), PCP-7 (), PCP-CA (), and PCP-3 (). Error bars represent standard deviation of respective parameters determined by the global fitting. Data for PCP-3 are from Ref. (28). PCP, procaspase.

Overall, the data suggest a minimum three-state process in all effector caspases in which a well-populated intermediate is in equilibrium with the native and unfolded protein. Comparatively, of the three human effector caspases and the CA, PCP-6 is the most stable with Δ*G*°_conf_ of 32.8 kcal/mol, PCP-CA and PCP-3 are intermediate with Δ*G*°_conf_ of 21.8 kcal/mol or 24.8 kcal/mol, respectively, and PCP-7 is the least stable with Δ*G*°_conf_ of 15.4 kcal/mol at pH 7.5 (Fig. 5).

### pH effects on equilibrium unfolding of effector caspases

We showed previously that PCP-3 undergoes pH-dependent conformational changes, and the dimer dissociates below pH 5.5 (28). From our previous data, we suggested that dimer dissociation was due to a series of salt bridges across the dimer interface, which include two histidine residues. The other effector caspases also have charged amino acids that interact across the dimer interface, but only PCP-3 contains the histidine residues. In order to determine the effects of pH on dimer stability, we performed equilibrium unfolding studies of PCP-6, PCP-7, and PCP-CA between pH 8.5 and pH 4. The data are summarized in Figure 5, and all folding/unfolding data are shown in Figures S2–S6, whereas the conformational stability and *m* value for each folding transition are shown in Tables S2 and S3.

**Figure 6:**
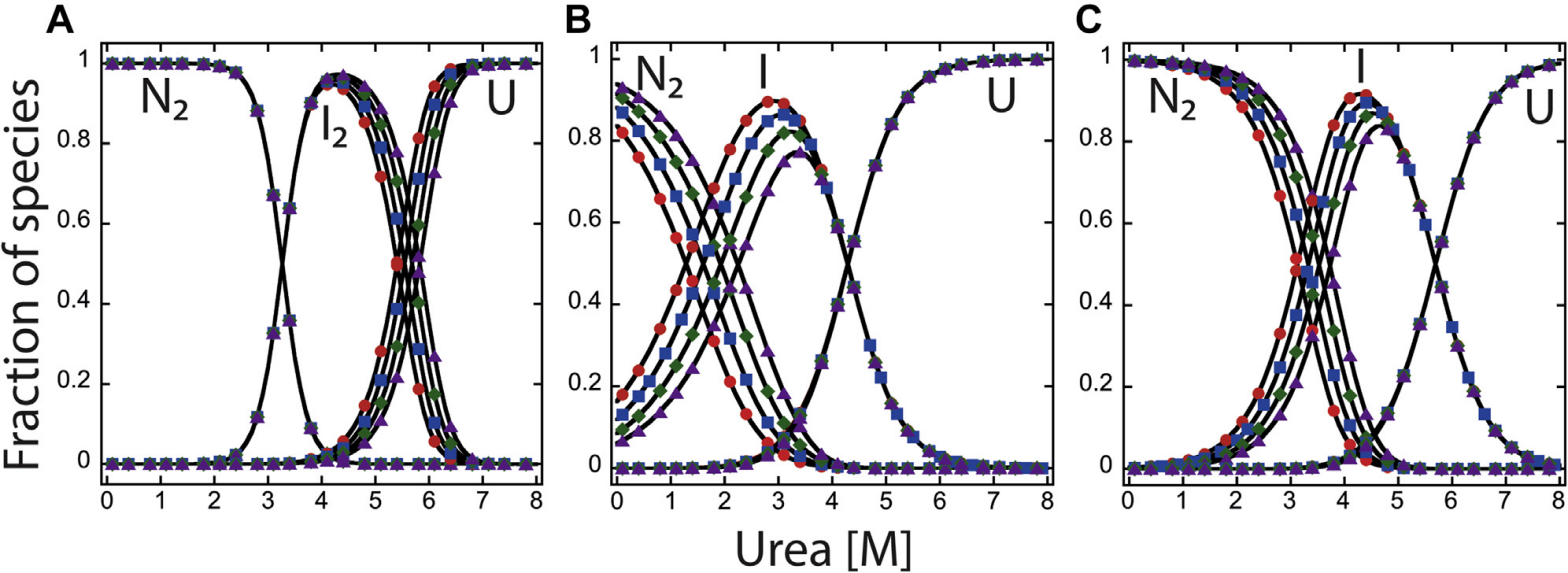
**Fraction of species as a function of urea concentration at pH 7.5.** Fraction of species of PCP-6 (*A*), PCP-7 (*B*), and PCP-CA (*C*). The fractions of native, intermediate, and unfolded protein were calculated as a function of urea concentration from fits of the data as shown in Figure 4. N_2_ refers to dimeric native protein, I_2_ and I are dimeric and monomeric intermediates, respectively, and U refers to unfolded species. The following protein concentrations were used: 0.5 μM (), 1 μM (), 2 μM (), and 4 μM (). PCP, procaspase.

Similarly to the data described previously at pH 7.5, the equilibrium folding/unfolding of PCP-6 can be described by a three-state equilibrium model (Equation 1) over the pH range of 4.5 to 8.5. At pH 4, however, the data were best described by a two-state equilibrium model where the native dimer is in equilibrium with the unfolded monomer (Equation 3) (Figs. S2 and S3). Surprisingly, even at pH 4, PCP-6 remains in a dimeric form, as demonstrated by the protein concentration dependence to unfolding (Figs. S2–S4). The protein stability, Δ*G*°_conf_, is ∼30 kcal/mol throughout the entire pH range (Fig. 5), demonstrating the consistently high conformational free energy of the PCP-6 dimer over a broad pH range. Based on the fits of the data to the corresponding equilibrium folding model (Tables S2 and S3), we calculated the fraction of species *versus* urea concentration at each pH examined (Figs. 6 and S4). The data show that the dissociation of the dimer is relatively consistent, with urea_½_ ∼6 M throughout the entire pH range. In contrast, the fraction of native dimer, N_2_, decreases below pH 6 relative to the fraction of dimeric intermediate, I_2_, such that at pH 4 the dimeric intermediate is fully populated in the absence of urea (Fig. S4).

In the case of PCP-7, the equilibrium folding/unfolding data were well described by the three-state equilibrium model, discussed previously for data at pH 7.5, in which the native dimer is in equilibrium with a monomeric intermediate (Equation 2) (Fig. S5). The conformational free energy (Δ*G*°_conf_) was 15 to 18 kcal/mol at higher pH (Tables S2 and S3), similar to the data described previously for pH 7.5. In contrast to PCP-6, however, we were unable to examine the equilibrium folding/unfolding of PCP-7 from pH 4.5 to pH 7 because protein aggregation resulted in irreversible unfolding. At pH 4, unfolding for PCP-7 is reversible, and the data were also best fit to the three-state equilibrium model with a monomeric intermediate (Equation 2). The data suggest that the primary differences in the fluorescence emission at pH 8 *versus* pH 4 are that the monomeric intermediate exhibits a higher fluorescence emission relative to the native conformation at pH 8. At pH 4, however, the fluorescence emission of the intermediate is lower than that of the native conformation. Overall, the data show that, while the overall conformational stability of PCP-7 is lower than that of PCP-6, at all pH, the unfolding of the monomeric intermediate has a similar urea_½_ of ∼4.5 M. In contrast, the native dimer is less stable at lower pH, although the protein appears to remain dimeric, and one observes that Δ*G*°_conf_ decreases by ∼5 kcal/mol because of the lower dimer stability (Table S2).

Similar to PCP-7, we observed that PCP-CA does not fold reversibly from pH 4.5 to pH 6 because of aggregation. The data for PCP-CA at pH 6.5 to pH 8.5 were best described by a three-state equilibrium model in which a monomeric intermediate is in equilibrium with the native dimer and unfolded monomer (Equation 2) (Figs. 4, *G*–*I* and S6), like PCP-7. One observes a protein concentration dependence to unfolding between ∼2 M and 5 M urea followed by a protein concentration–independent transition between ∼5 M to 7 M urea. Based on global fits of the data to the three-state equilibrium model (Equation 2), we determined that the total conformational free energy on unfolding of PCP-CA from pH 6.5 to pH 8.5 is ∼20 kcal/mol (Fig. 5 and Tables 1, S2, and S3). In contrast, the equilibrium folding/unfolding data at pH 4 were the best fit to a three-state model in which the native monomer unfolds through a monomeric intermediate (Equation 4). We noted that we cannot rule out that a small population of dimer is present at pH 4, but fitting the data to include a native dimer (Equation 2), dimeric intermediate (Equation 1), or no intermediate did not improve the quality of the fits. Our interpretation of the results is that the Δ*G*°_conf_ of the native species at pH 4 is very low (0.4 kcal/mol; see Table S2), so it is difficult to observe a protein concentration dependence to unfolding. A comparison of the changes in secondary structure observed by CD effectively illustrates the point, when compared at pH 8 *versus* pH 4 (compare Figs. S6, *C* and *G*). Thus, we selected the simplest model to describe the data, Equation 4, which suggests that PCP-CA is a monomer at pH 4 and unfolds through a partially folded monomeric intermediate. In this case, the Δ*G*°_conf_ of unfolding is 5.5 kcal/mol (Fig. S6 and Tables S2 and S3).

The total conformational free energies of unfolding, Δ*G*°_conf_, and *m* values obtained from the fits of all three proteins over the pH range of 4 to 8.5 are shown in Figure 5, *A* and *B*, respectively. Because of aggregation upon refolding of PCP-CA at pH range 4.5 to 6, we were unable to determine the precise pH range for the transition of dimer to monomer. Nevertheless, PCP-CA is similar to PCP-3 (28) in that the dimer is destabilized at lower pH, such that the protein is a monomer at pH 4. Together, the data show that the dimer is destabilized in PCP-3 and PCP-7 at lower pH, although PCP-7, like PCP-6, remains dimeric. Because the dimer contributes a substantial portion of the conformational free energy, PCP-6 and PCP-7 exhibit a relatively consistent Δ*G*°_conf_ at all pHs, whereas the Δ*G*°_conf_ of PCP-3 and PCP-CA reflect the stability of the monomer at pH 4 (Fig. 5). Unlike PCP-3, however, we were unable to determine the p*K*a for dimer dissociation of PCP-CA because of protein aggregation between pH 4.5 and 6.5.

From the global fitting, we also calculated the cooperativity index (*m* value) for each unfolding step. The *m* value relates to the accessible surface area (ΔASA) exposed to solvent during unfolding (31). The total *m* value (*m*_total_) of PCP-6, PCP-7, and PCP-CA range from 2.5 to 5.5 kcal mol^−1^ M^−1^, respectively, at higher pH (Fig. 5*B* and Tables 1 and S3). Scholtz *et al.* (31) developed an empirical analysis to show the correlation of ΔASA with experimental *m* values (Equation 5). Using their analysis, our data suggest that the native dimers of PCP-7 and PCP-CA are less compact than those of PCP-6 or PCP-3. One observes that the *m* values of the PCP-7 and PCP-CA monomers are similar to that determined previously for PCP-3 (∼1.2 kcal mol^−1^ M^−1^) (Table S3), suggesting that the surface area exposed during unfolding of the monomer is similar for the effector caspases. In contrast, the native dimer of PCP-7 and PCP-CA appear to have larger exposed surface area compared with those of PCP-3 or PCP-6, resulting in a lower *m* value (and related ΔASA) during unfolding of the dimer.

### The fraction of species *versus* urea concentration

For each pH, we calculated the equilibrium distribution of species over the urea concentration range of 0 to 8 M using the values obtained from the global fits of the equilibrium unfolding data (described previously), the cooperativity indices determined for each transition (Tables S2 and S3), and four protein concentrations (0.5, 1, 2, and 4 μM). The fractions of species for PCP-6, PCP-7, and PCP-CA are shown in Figures 6 and S4–S6. Collectively, at pH 7.5, one observes a cooperative decrease in native dimer (N_2_) with a concomitant increase in a partially folded intermediate between 0 and ∼4 M urea (Fig. 6). In the case of PCP-6 and PCP-3 (28), the intermediate is dimeric (I_2_), where for PCP-7 and PCP-CA, the intermediate is a monomer (I). The dimeric (PCP-6 or PCP-3) or monomeric (PCP-7 or PCP-CA) intermediate reaches a maximum at ∼3 to 4 M urea and remains predominant to ∼5 to 6 M urea (Figs. 6 and S4–S6). The unfolded state is fully populated by ∼7 M urea in all cases. At pH 4, the “native” ensemble of PCP-6 and PCP-7 consists of a dimeric conformation (Figs. S4*A* and S5*G*), whereas the major fraction of PCP-CA (Fig. S6*D*) consists of monomers. Together, the pH studies suggest that the native dimer (N_2_) of the effector caspases (PCP-6, PCP-3, and PCP-7) and the CA (PCP-CA) is destabilized at low pH relative to a partially folded intermediate, either the dimer, I_2_ (PCP-6), or the monomer, I (PCP-7, PCP-3, and PCP-CA).

Previously, we showed that the dimer of PCP-3 undergoes a pH-dependent conformational change and that the p*K*a for the transition is ∼5.7 (32). Likewise, the data for PCP-6 suggest that a similar transition occurs in the dimer, with a similar p*K*a of ∼5.9 (Fig. S4). In this case, we examined the midpoint of the first transition at each pH (N_2_ ⇄ I_2_). In PCP-3, however, a second transition occurs as the pH is lowered such that the dimer dissociates, with a p*K*a ∼4.7. Thus, PCP-6 and PCP-3 undergo a similar pH-dependent transition of N_2_ to I_2_, with p*K*a ∼5.7 to 5.9, but PCP-6 remains dimeric at lower pH, whereas the dimer of PCP-3 dissociates.

## Discussion

The caspase–hemoglobinase fold is an ancient protein fold that has been conserved for at least 650 million years (15) and from which evolved three subfamilies of caspases (13). All caspases are produced in the cell as inactive zymogens that must be activated prior to their function in the inflammatory response or in apoptosis. The effector caspase zymogens are unique among the caspase subfamilies in that the proteins are stable, yet inactive, dimers (13). In addition to differences in oligomeric states, the caspase–hemoglobinase fold is also the basis for the evolution of separate enzyme specificity and allosteric regulation (33, 34). However, the evolutionary processes that resulted in enzymatic, allosteric, and oligomeric diversity of the caspases are unknown. Effector caspase-3, caspase-6, and caspase-7 have a significant role in apoptosis and serve overlapping but nonredundant functions (35), and multiple studies have examined enzyme specificity and regulation of extant caspases (21, 33, 36, 37). In addition, we have previously examined evolutionary changes resulting in amino acid substitutions that affect enzyme specificity (15, 38) and allosteric regulation (26, 39, 40), but there is a dearth of information regarding changes in the caspase folding landscape. To date, only human caspase-3 has been examined in detail (6, 27, 28, 41), so it was not clear whether all effector caspases utilized the same folding landscape.

Here, we examined the conservation of the folding landscape by determining the equilibrium folding/unfolding process of the CA of effector caspases as well as human PCP-6 and PCP-7, and we compared the results to our previous data for human PCP-3. Collectively, the data provide a baseline for understanding the folding landscape of effector caspases and serve as a platform for examining the monomeric caspase subfamilies as well as evolutionary changes that resulted in the stable dimer. The PCP-3 dimer folds and assembles through two partially folded intermediate conformations, a monomer (I) and a dimer (I_2_), with an overall conformational free energy, Δ*G*°_conf_, of ∼22 kcal/mol at pH >6 (27, 28). Our data for the folding and assembly of the CA of effector caspases, PCP-CA, show that the monomeric intermediate (I) is present in the folding pathway, but we do not observe the dimeric intermediate (I_2_). In contrast, our data for the folding and assembly of the PCP-6 dimer show that the dimeric intermediate (I_2_) is present in the folding pathway, but we do not observe the monomeric intermediate (I). Finally, PCP-7 is more similar to the CA, with the monomeric intermediate (I) present in the folding pathway. We suggest that a parsimonious explanation for the data is that the effector caspase folding landscape consists of both intermediates, I and I_2_, and that the population distribution of I relative to I_2_ varies among the three extant caspases. In this case, the more prevalent intermediate, I or I_2_, is observed in our spectroscopic assays. For PCP-3, both intermediates are observed because of their similar stability, resulting in a substantial population of both species. We note that our data do not rule out the possibility that the dimeric intermediate arose separately in PCP-3 and PCP-6 after the three lineages split ∼450 million years ago. Nevertheless, such a folding landscape, where both intermediates are accessible for evolutionary changes, would provide flexibility in a protein fold that is utilized by multiple subfamilies. In this case, amino acid changes that occur through evolution could stabilize one intermediate relative to the other in a species-dependent manner to provide for differences in cellular and environmental conditions of the organism. Our equilibrium data presented here do not provide information on the rate of folding, so further kinetic studies would determine whether changes in the relative distribution of I and I_2_ affect folding efficiency. We note, however, that mutations in PCP-3 that affect the rate of I-to-I_2_ dimerization did result in formation of a dimerization-incompetent monomer, leading to hysteresis in the equilibrium folding data (41).

Although the folding landscape of caspases has been conserved for >650 million years, amino acid substitutions through evolution have resulted in differences in stability among the extant caspases. When comparing the overall conformational stability of the extant human effector caspases, one observes that the dimer generally falls in the range of ∼17 to 30 kcal/mol (Table S2). PCP-6 is the most stable, with Δ*G*°_conf_ of ∼30 kcal/mol at pH >6, and PCP-7 is the least stable, with Δ*G*°_conf_ of ∼17 kcal/mol at pH >6. PCP-3 and PCP-CA show similar stabilities, with Δ*G*°_conf_ of ∼22 kcal/mol at pH >6. The unfolding data also show that the dimer of PCP-6 is more stable than that of PCP-7 or PCP-CA, where the midpoint of dimer dissociation is approximately 5 M urea for PCP-6 *versus* 2 M urea for PCP-7 and PCP-CA, likely because of the higher population of the I_2_ intermediate in PCP-6 compared with PCP-7 and PCP-CA. Notably, Matthews *et al.* (42, 43) have reported that amino acid substitutions that change in the number of salt bridges in the native structure of a protein are a key parameter in modulating the free energy landscape of a protein fold. We compared the number of salt bridges among extant caspases using a protein tool developed by Ferruz *et al.* (44). The analysis showed that PCP-3 (Protein Data Bank [PDB]: 1CP3), PCP-6 (PDB: 3S70), and PCP-7 (PDB: 1F1J) consist of 21, 22, and 15 salt bridges, respectively. Although the data are consistent with PCP-7 exhibiting the lowest conformational free energy, further examination of the intersubunit contacts would provide a quantitative assessment of the contributions to overall stability.

**Figure 7:**
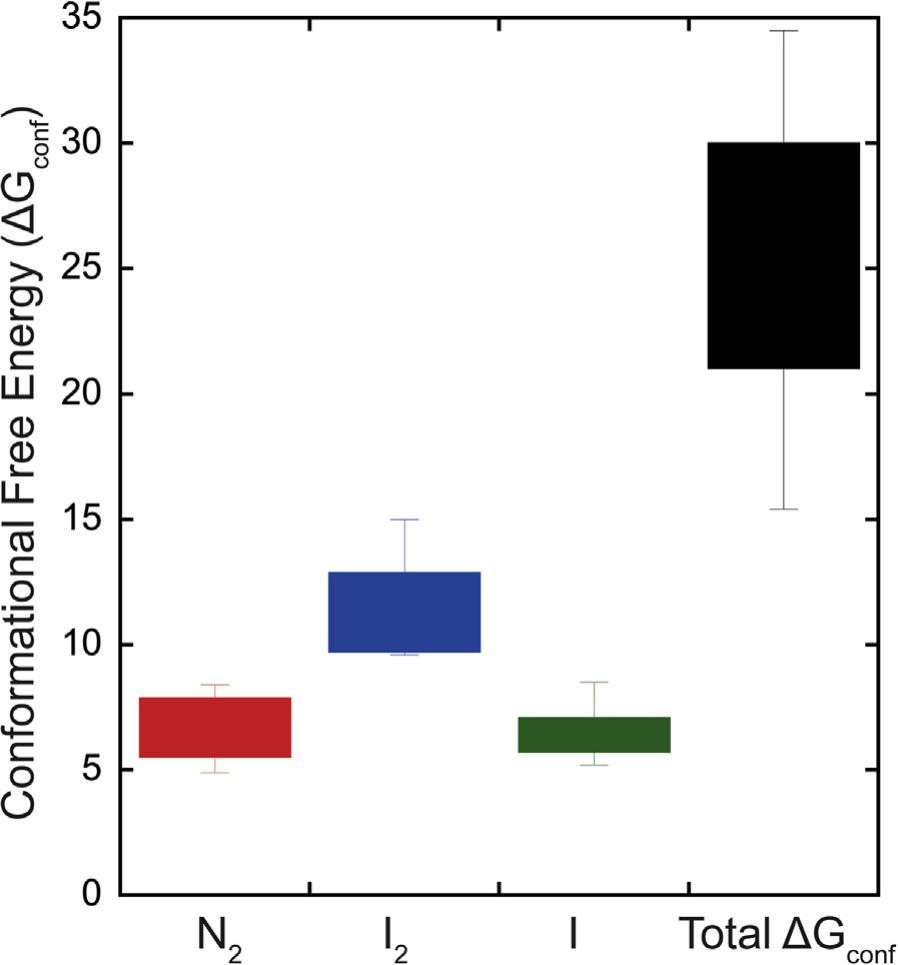
**Conformational free energies of three conformations (N**_**2**_**, I**_**2**_**, and I) in the caspase folding landscape as well as the total conformational free energy for effector caspases.** Values were calculated as described in the text using data in Table S2.

Our data show that the conformational stability is similar for the different species in the folding landscape, so based on the data for the four effector caspases, we compared the Δ*G*°_conf_ for each transition to determine a range of conformational free energy for the native dimer (N_2_), the dimeric intermediate (I_2_), and the monomeric intermediate (I) (Fig. 7). We used Δ*G*°_conf_ values at the higher pHs since three of the caspases (PCP-3, PCP-7, and PCP-CA) were less stable at low pH. Essentially, the values of $\Delta G_{1}^{{\text{H}}_{2}\text{O}}$, $\Delta G_{2}^{{\text{H}}_{2}\text{O}}$, and $\;\Delta G_{3}^{{\text{H}}_{2}\text{O}}$from Table S2 were utilized in the analysis. The comparison shows that the native dimer of effector caspases has a Δ*G*°_conf_ of ∼6.7 kcal/mol with a range of 4.9 to 8.7 kcal/mol. The dimeric intermediate is more stable, with Δ*G*°_conf_ of ∼11.3 kcal/mol, within a range of 9.5 to 15 kcal/mol, and the monomeric intermediate is similar to that of the dimer, with Δ*G*°_conf_ of ∼6.4 kcal/mol, within a range of 5.2 to 8.5 kcal/mol. Combining the three species results in the overall conformational free energy of 25.7 kcal/mol, with a broad range of 15 to 34 kcal/mol. We suggest that the folding landscape of effector caspases was established in the CA and provides two partially folded conformations, one monomer and one dimer, from which evolutionary changes can establish the relative distribution of the intermediates as well as the overall conformational stability of the dimer, within the ranges shown in Figure 7.

We showed previously that changes in pH are an excellent perturbation of the caspase folding landscape because the PCP-3 dimer dissociates below pH 5 such that the protein is a monomer at pH 4 (28). Furthermore, we suggested that two histidine residues in PCP-3 stabilize the dimer at higher pH through charge–charge interactions across the dimer interface. In contrast to PCP-3, PCP-6 remains dimeric over the entire pH range examined here (pH 4–8.5), and we note that CP-H186 is unique in PCP-3 (Fig. 2). At the CP-186 position, one observes glutamate in PCP-6 and PCP-CA and glutamine in PCP-7. In addition, the PCP-7 dimer is less stable at lower pH, but the protein remains dimeric. At present, it is not clear why the PCP-CA is less stable at lower pH, but the data suggest that evolutionary changes stabilized the effector caspase dimer against pH changes, except for PCP-3. We noted previously (28) that pH changes in the cell during apoptosis (45, 46) may affect the monomer–dimer equilibrium of caspase-3, resulting in lower activity overall for the pool of caspase-3 in the apoptotic cell, since the monomer is enzymatically inactive. In this case, the pool of caspase-6 and/or caspase-7 activity would be affected only by the protonation/deprotonation of the catalytic cysteine–histidine residues rather than the additional regulatory feature of monomer–dimer equilibrium. Since PCP-7 and PCP-CA were unfolded irreversibly around pH 5 and 6, it is not clear at what pH the protein dissociates to monomers or why the dimer is less stable at lower pH. Overall, however, the differences in pH effects suggest that caspase-6 could be the main executioner caspase in a low pH environment rather than caspase-3. Caspase-6 has a more specialized role in specific physiological contexts compared with caspase-3 and caspase-7 (47, 48, 49). Further analysis of the differences in the dimeric interface among effector caspases would provide clarity regarding the evolutionary changes that resulted in the higher conformational stability of the PCP-6 dimer at low pH.

In summary, by comparing the equilibrium folding/unfolding of the human effector caspases and their CA, we show that the folding landscape was established in the CA (>650 million years ago) and that evolution can utilize intermediates in the landscape to effect changes in the conformational stability of extant caspases, within the constraints of the caspase–hemoglobinase fold. The caspase family highlights how sequence changes through evolutionary processes can provide species-dependent flexibility in the caspase dimer. Hence, the stability of the native enzyme and the response to changes in the environment can be fine-tuned in a species-specific manner while retaining the overall caspase–hemoglobinase fold.

## Experimental procedures

### Cloning, protein expression, and protein purification

For all PCP proteins, the catalytic cysteine (CP-117; Fig. 2) was mutated to serine using site-directed mutagenesis, as described previously (50). The inactive PCPs were cloned into the pET11a expression vector with a C-terminal hexahistidine tag, and all proteins were expressed in *E. coli* BL21(DE3) pLysS cells and purified as previously described (32, 51, 52).

### Sample preparation for equilibrium unfolding

Denaturation and renaturation experiments were carried out as described previously (53). Briefly, urea stock solutions (10 M) were prepared in citrate buffer (20 mM sodium citrate/citric acid, pH 4 to pH 5.5, 1 mM DTT), phosphate buffer (20 mM potassium phosphate monobasic and dibasic, pH 6–8, 1 mM DTT), or Tris buffer (20 mM Tris–HCl, pH 8.5, 1 mM DTT). For unfolding experiments, samples were prepared in the corresponding buffer with final urea concentrations between 0 and 8 M. Stock protein in buffer was added such that the final concentrations are as shown in the figures. For renaturation experiments, the protein was first incubated in an 8 M urea-containing buffer for 3 h at 25 °C. The unfolded protein was then diluted with the corresponding buffer and urea such that the final urea concentrations were between 0.5 and 8 M. For all equilibrium unfolding experiments, protein concentrations from 0.5 to 4 μM were used. In both denaturation and renaturation experiments, the samples were incubated at 25 °C for a minimum of 16 h to allow for equilibration. This incubation time was found to be optimal to allow the protein to reach equilibrium at all urea concentrations.

### Fluorescence emission and CD measurements

Fluorescence emission was acquired using a PTI C-61 spectrofluorometer (Photon Technology International) from 300 to 400 nm following excitation at 280 or 295 nm. Excitation at 280 nm follows tyrosinyl and tryptophanyl fluorescence emission, whereas excitation at 295 nm follows the tryptophanyl fluorescence emission. CD measurements were recorded using a J-1500 CD spectropolarimeter (Jasco) between 220 and 240 nm. Fluorescence and CD spectra were measured using a 1-cm path length cuvette and constant temperature (25 °C). All data were corrected for buffer background.

### Data analysis and global fits of the equilibrium unfolding data

The data were fit globally and interpreted as described previously (27, 28, 53). Briefly, fluorescence emission and CD data were collected between pH 8.5 and 4 for all three proteins and at three to four protein concentrations, which resulted in 9 to 12 datasets at each pH. The data were fit to a two-state or three-state equilibrium folding model, as described later. At pH 7.5, the data for PCP-6 were best fit to a three-state equilibrium folding model described with a dimeric intermediate in equilibrium with the native and unfolded protein, as shown in Equation 1. In contrast, the data for PCP-7 and PCP-CA were best fit to a three-state equilibrium folding model described with a monomeric intermediate in equilibrium with the native and unfolded protein, as shown in Equation 2.

(1) $$N_{2}\;\overset{K_{1}}{\Leftrightarrow }I_{2}\;\overset{K_{2}}{\Leftrightarrow }\;2U$$

(2) $$N_{2}\;\overset{K_{1}}{\Leftrightarrow }\;2I\overset{K_{2}}{\Leftrightarrow }\;2U$$

In both Equations (1), (2) and (1), (2), *K*_1_ and *K*_2_ refer to equilibrium constants for the two steps, respectively. At pH 4, the data for PCP-6 were best fit to a two-state equilibrium folding model, where the native dimer is in equilibrium with the unfolded protein, as shown in Equation 3.

(3) $$N_{2}\;\overset{K_{eq}}{\Leftrightarrow }\;2U$$

In contrast to PCP-6, at pH 4, the data for PCP-7 were best fit to a three-state equilibrium folding model for a monomer, as shown in Equation 4.

(4) $$N\overset{K_{1}}{\Leftrightarrow }I\overset{K_{2}}{\Leftrightarrow }\;U$$

For all proteins, the equilibrium folding/unfolding data at each pH were fit globally using the appropriate folding model from Equations (1), (2), (3), (4), (5) and the program Igor Pro (WaveMetrics, Inc), as described previously (27, 28, 53). Results from the fits are shown in Table 1 and as the solid lines in Figures 4 and S2–S6. The change in solvent ASA was calculated as described by Scholtz *et al.* (31), as shown in Equation 5.

(5) m = 243 + 0.13(ΔASA)

## Data availability

All data are contained in the article and supporting information.

## Supporting information

This article contains supporting information (27, 28, 32).

## Conflict of interest

The authors declare that they have no conflicts of interest with the contents of this article.

## References

[1] Doyle C.M., Rumfeldt J.A., Broom H.R., Broom A., Stathopulos P.B., Vassall K.A., Almey J.J., Meiering E.M. (2013). Energetics of oligomeric protein folding and association. Arch. Biochem. Biophys..

[2] Rumfeldt J.A.O., Galvagnion C., Vassall K.A., Meiering E.M. (2008). Conformational stability and folding mechanisms of dimeric proteins. Prog. Biophys. Mol. Biol..

[3] Röder K., Joseph J.A., Husic B.E., Wales D.J. (2019). Energy landscapes for proteins: From single funnels to multifunctional systems. Adv. Theor. Simulations..

[4] Kentsis A., Gindin T., Mezei M., Osman R. (2007). Calculation of the free energy and cooperativity of protein folding. PLoS One.

[5] Hollien J., Marqusee S. (2002). Comparison of the folding processes of *T. thermophilus* and *E. coli* ribonucleases H. J. Mol. Biol..

[6] Milam S.L., Clark A.C. (2009). Folding and assembly kinetics of procaspase-3. Protein Sci..

[7] Yao J., Wang J. (2015). Neither two-state nor three-state: Dimerization of lambda cro repressor. J. Phys. Chem. Lett..

[8] Zheng W., Schafer N.P., Davtyan A., Papoian G.A., Wolynes P.G. (2012). Predictive energy landscapes for protein-protein association. Proc. Natl. Acad. Sci. U. S. A..

[9] Han J.H., Batey S., Nickson A.A., Teichmann S.A., Clarke J. (2007). The folding and evolution of multidomain proteins. Nat. Rev. Mol. Cell Biol..

[10] Lynch M. (2012). The evolution of multimeric protein assemblages. Mol. Biol. Evol..

[11] Lynch M. (2013). Evolutionary diversification of the multimeric states of proteins. Proc. Natl. Acad. Sci. U. S. A..

[12] Hashimoto K., Panchenko A.R. (2010). Mechanisms of protein oligomerization, the critical role of insertions and deletions in maintaining different oligomeric states. Proc. Natl. Acad. Sci. U. S. A..

[13] Clark A.C. (2016). Caspase allostery and conformational selection. Chem. Rev..

[14] MacKenzie S.H., Clark A.C. (2012). Death by caspase dimerization. Adv. Exp. Med. Biol..

[15] Grinshpon R.D., Shrestha S., Titus-McQuillan J., Hamilton P.T., Swartz P.D., Clark A.C. (2019). Resurrection of ancestral effector caspases identifies novel networks for evolution of substrate specificity. Biochem. J..

[16] Aravind L., Koonin E.V. (2002). Classification of the caspase–hemoglobinase fold: Detection of new families and implications for the origin of the eukaryotic separins. Proteins.

[17] Lim S.A., Marqusee S. (2018). The burst-phase folding intermediate of ribonuclease H changes conformation over evolutionary history. Biopolymers.

[18] Lim S.A., Hart K.M., Harms M.J., Marqusee S. (2016). Evolutionary trend toward kinetic stability in the folding trajectory of RNases H. Proc. Natl. Acad. Sci. U. S. A..

[19] Pillai A.S., Chandler S.A., Liu Y., Signore A.V., Cortez-Romero C.R., Benesch J.L.P., Laganowsky A., Storz J.F., Hochberg G.K.A., Thornton J.W. (2020). Origin of complexity in haemoglobin evolution. Nature.

[20] Harms M.J., Thornton J.W. (2013). Evolutionary biochemistry: Revealing the historical and physical causes of protein properties. Nat. Rev. Genet..

[21] Fuentes-Prior P., Salvesen G.S. (2004). The protein structures that shape caspase activity, specificity, activation and inhibition. Biochem. J..

[22] Tucker M.B., MacKenzie S.H., Maciag J.J., Dirscherl A.H., Swartz P., Yoder J.A., Hamilton P.T., Clark A.C. (2016). Phage display and structural studies reveal plasticity in substrate specificity of caspase-3a from zebrafish. Protein Sci..

[23] Feeney B., Pop C., Swartz P., Mattos C., Clark A.C. (2006). Role of loop bundle hydrogen bonds in the maturation and activity of (Pro)caspase-3. Biochemistry.

[24] Huang W., Jiang T., Choi W., Qi S., Pang Y., Hu Q., Xu Y., Gong X., Jeffrey P.D., Wang J., Shi Y. (2013). Mechanistic insights into CED-4-mediated activation of CED-3. Genes Dev..

[25] Yan N., Huh J.R., Schirf V., Demeler B., Hay B.A., Shi Y. (2006). Structure and activation mechanism of the Drosophila initiator caspase Dronc. J. Biol. Chem..

[26] Shrestha S., Tung J., Grinshpon R.D., Swartz P., Hamilton P.T., Dimos B., Mydlarz L., Clark A.C. (2020). Caspases from scleractinian coral show unique regulatory features. J. Biol. Chem..

[27] Bose K., Clark A.C. (2001). Dimeric procaspase-3 unfolds via a four-state equilibrium process. Biochemistry.

[28] Bose K., Clark A.C. (2005). pH effects on the stability and dimerization of procaspase-3. Protein Sci..

[29] Merkl R., Sterner R. (2016). Reconstruction of ancestral enzymes. Perspect. Sci..

[30] Lakowicz J.R. (2006). Principles of Fluorescence Spectroscopy.

[31] Scholtz J.M., Grimsley G.R., Pace C.N. (2009). Solvent denaturation of proteins and interpretations of the m value. Methods Enzymol..

[32] Bose K., Pop C., Feeney B., Clark A.C. (2003). An uncleavable procaspase-3 mutant has a lower catalytic efficiency but an active site similar to that of mature caspase-3. Biochemistry.

[33] Dagbay K., Eron S.J., Serrano B.P., Velázquez-Delgado E.M., Zhao Y., Lin D., Vaidya S., Hardy J.A. (2014). A multipronged approach for compiling a global map of allosteric regulation in the apoptotic caspases. Methods Enzymol..

[34] Hill M.E., Macpherson D.J., Wu P., Julien O., Wells J.A., Hardy J.A. (2016). Reprogramming caspase-7 specificity by regio-specific mutations and selection provides alternate solutions for substrate recognition. ACS Chem. Biol..

[35] Thomsen N.D., Koerber J.T., Wells J.A. (2013). Structural snapshots reveal distinct mechanisms of procaspase-3 and -7 activation. Proc. Natl. Acad. Sci. U. S. A..

[36] MacPherson D.J., Mills C.L., Ondrechen M.J., Hardy J.A. (2019). Tri-arginine exosite patch of caspase-6 recruits substrates for hydrolysis. J. Biol. Chem..

[37] McIlwain D.R., Berger T., Mak T.W. (2013). Caspase functions in cell death and disease. Cold Spring Harb. Perspect. Biol..

[38] Kumar S., Van Raam B.J., Salvesen G.S., Cieplak P. (2014). Caspase cleavage sites in the human proteome: CaspDB, a database of predicted substrates. PLoS One.

[39] Maciag J.J., Mackenzie S.H., Tucker M.B., Schipper J.L., Swartz P., Clark A.C. (2016). Tunable allosteric library of caspase-3 identifies coupling between conserved water molecules and conformational selection. Proc. Natl. Acad. Sci. U. S. A..

[40] Thomas M.E., Grinshpon R., Swartz P., Clark A.C. (2018). Modifications to a common phosphorylation network provide individualized control in caspases. J. Biol. Chem..

[41] Mackenzie S.H., Clark A.C. (2013). Slow folding and assembly of a procaspase-3 interface variant. Biochemistry.

[42] Basak S., Paul N.R., Tavella D., Deveau L.M., Koga N., Tatsumi-Koga R., Baker D., Massi F., Robert M.C. (2019). Networks of electrostatic and hydrophobic interactions modulate the complex folding free energy surface of a designed βα protein. Proc. Natl. Acad. Sci. U. S. A..

[43] Kleiner D., Shmulevich F., Zarivach R., Shahar A., Sharon M., Ben-Nissan G., Bershtein S. (2019). The interdimeric interface controls function and stability of *Ureaplasma urealiticum* methionine S-adenosyltransferase. J. Mol. Biol..

[44] Ferruz N., Schmidt S., Höcker B. (2021). ProteinTools: A toolkit to analyze protein structures. Nucleic Acids Res..

[45] Gottlieb R.A., Nordberg J., Skowronski E., Babior B.M. (1996). Apoptosis induced in Jurkat cells by several agents is preceded by intracellular acidification. Proc. Natl. Acad. Sci. U. S. A..

[46] Matsuyama S., Llopis J., Deveraux Q.L., Tsien R.Y., Reed J.C. (2000). Changes in intramitochondrial and cytosolic pH: Early events that modulate caspase activation during apoptosis. Nat. Cell Biol..

[47] Ehrnhoefer D.E., Skotte N.H., Reinshagen J., Qiu X., Windshügel B., Jaishankar P., Ladha S., Petina O., Khankischpur M., Nguyen Y.T.N., Caron N.S., Razeto A., Meyer zu Rheda M., Deng Y., Huynh K.T. (2019). Activation of caspase-6 is promoted by a mutant Huntingtin fragment and blocked by an allosteric inhibitor compound. Cell Chem. Biol..

[48] Yao Y., Shi Q., Chen B., Wang Q., Li X., Li L., Huang Y., Ji J., Shen P. (2016). Identification of caspase-6 as a new regulator of alternatively activated macrophages. J. Biol. Chem..

[49] Slee E.A., Adrain C., Martin S.J. (2001). Executioner caspase-3, -6, and -7 perform distinct, non-redundant roles during the demolition phase of apoptosis. J. Biol. Chem..

[50] Pop C., Chen Y.R., Smith B., Bose K., Bobay B., Tripathy A., Franzen S., Clark A.C. (2001). Removal of the pro-domain does not affect the conformation of the procaspase-3 dimer. Biochemistry.

[51] MacKenzie S.H., Schipper J.L., England E.J., Thomas M.E., Blackburn K., Swartz P., Clark A.C. (2013). Lengthening the intersubunit linker of procaspase 3 leads to constitutive activation. Biochemistry.

[52] Roschitzki-Voser H., Schroeder T., Lenherr E.D., Frölich F., Schweizer A., Donepudi M., Ganesan R., Mittl P.R.E., Baici A., Grütter M.G. (2012). Human caspases *in vitro*: Expression, purification and kinetic characterization. Protein Expr. Purif..

[53] Walters J., Milam S.L., Clark A.C. (2009). Practical approaches to protein folding and assembly: Spectroscopic strategies in thermodynamics and kinetics. Methods Enzymol..

[54] Grinshpon R.D., Williford A., Titus-McQuillan J., Clark A.C. (2018). The CaspBase: A curated database for evolutionary biochemical studies of caspase functional divergence and ancestral sequence inference. Protein Sci..

